# Insights into Catechin–Copper Complex Structure and Biologic Activity Modulation

**DOI:** 10.3390/molecules29204969

**Published:** 2024-10-21

**Authors:** Ionut I. Lungu, Oana Cioanca, Cornelia Mircea, Cristina Tuchilus, Alina Stefanache, Riana Huzum, Monica Hancianu

**Affiliations:** 1 Faculty of Pharmacy, “Grigore T. Popa” University of Medicine and Pharmacy, 700115 Iasi, Romania; 2 Faculty of Medicine, “Grigore T. Popa” University of Medicine and Pharmacy, 400347 Iasi, Romania

**Keywords:** copper–catechin complex, antioxidant activity, antibacterial

## Abstract

Compounds of natural origin found in varying quantities in plant-based products constitute a highly significant category, possessing structural significance as well as the capacity to regulate oxidative processes. The activity of these compounds may be modulated by the composition of the biological environment in which they operate, the pH of the environment, or the presence of metal cations in plants or plant extracts. A successful complexation reaction was mainly confirmed by FT-IR, observing the shift from the original transmittance of catechin bonds, especially O-H ones. This work shows the synthetic methodology and the optimization process that took place to synthesize a catechin–copper complex, which demonstrated antioxidant activity. It was tested for iron chelation ability, hydroxyl radicals, and the inhibition of lipoxygenase (15-LOX). An antidiabetic assay was performed by determining the inhibition of alpha-amylase and alpha-glucosidase, finding that the synthesized complex had similar inhibitory potential as pure catechin. The antibacterial tests showed results against *Staphylococcus aureus* and the antifungal properties of the complex against *Candida albicans*.

## 1. Introduction

Metal chelation is an important property of flavonoids, and it holds considerable importance due to its profound influence on the magnitude of the pharmacological effects that flavonoids can exert by regulating the bioavailability of diverse minerals and ions. Catechins are a class of natural polyphenolic compounds known as flavanols, which are members of the flavonoid family. Abundant concentrations of these compounds can be found in a diverse range of fruits, vegetables, and plant-based beverages. The term “catechin” is etymologically derived from the scientific name of the Cutch tree, known as *Acacia catechu L.f.* Fresh tea leaves, rock-rose leaves, broad beans, red wine, black grapes, strawberries, and apricots are known to contain elevated levels of catechin [1,2,3].

Certain flavonoids have been identified to possess the ability to chelate plurivalent metals such as iron, zinc, and copper. According to [4], catechin has the ability to mitigate the harmful consequences of oxidation on the erythrocyte membrane. This is achieved by counteracting the impact of various oxidizing agents that trigger the liberation of iron in its free, redox-active state. The binding sites of metals to flavonoids are located in the B ring, at the 3-hydroxyl and 4-oxo groups in the heterocyclic ring C, as well as at the 4-oxo and 5-hydroxyl groups between the C and A groups [5,6,7].

Chelates, which are complexes formed by flavonoids with metal ions, exhibit significant effects in terms of their antioxidative, antibacterial, and antidiabetic activities. Multiple studies have demonstrated that the anti-radical properties of flavonoid–metal complexes are more potent compared to flavonoids themselves. Kostyuk et al. (2001) [8] conducted a study that provided evidence for the enhanced efficacy of rutin, quercetin, and catechin when complexed with Fe (II), Fe (III), Cu (II), and Zn (II) compared to their free forms. This increased effectiveness can be attributed to the incorporation of an additional dismutation center [9,10,11]. 

The human body possesses a significant array of defensive mechanisms to protect itself from the detrimental effects of oxidative stress. Enzymes, namely superoxide dismutase (SOD), catalase (CAT), and glutathione peroxidase (Gpx), are integral components of the cellular defense mechanism against reactive oxygen species (ROS). These enzymes are further aided by the activities of glutathione reductase (GR) and glucose-6-phosphate dehydrogenase. Copper (Cu) is a constituent of cytosolic superoxide dismutase (SOD), while manganese (Mn) is a component of mitochondrial SODs. Additionally, there exist extracellular superoxide dismutases (SODs). The Cu/Zn plasma ratio holds significance in relation to SOD activity, as a reduction in enzymatic cofactors results in a decline in SOD activity [12]. The antioxidant capacities of flavonoids exhibit a notable superior activity in comparison to those of vitamins. Flavonoids possess the capability to directly counteract free radicals through the process of hydrogen atom donation. The biological activities of flavonoids in an in vitro setting are contingent upon the arrangement of functional groups within their molecular structure. According to [8], antioxidant activity is consistently influenced by the arrangement and quantity of hydroxyl groups.

The present study contributes to an enhanced comprehension of the factors that dictate the associations between the molecular structure of flavonoids and their capacity to engage in metal complexation, and it is focused on understanding the complex synthesis and to fully understand the antibacterial, antioxidant, and antidiabetic activities of the synthesized copper–catechin complex [13].

## 2. Results and Discussions

### 2.1. Synthesis and Complexation Yield

The primary parameters evaluated in this study included adjusting the different ratios of reagents, the choice of solvent for achieving optimal yield, the control of reaction temperature, and the adjustment of the pH solution in order to encourage precipitate formation and improve the yield of the reaction. The resulting precipitate was confirmed to be the desired complex, after which we determined the yield of the reaction by weighing the dry precipitate.

Furthermore, the stirring velocity and duration were modified to promote an enhanced interaction between the main reagents (complexing agents: catechin and copper salt) and the solvent. Table 1 presents the reaction yields achieved by varying these parameters. Despite our attempts to modulate the reaction medium through pH adjustments, the most intriguing outcomes were observed when the pH was brought up to 8.5 utilizing a 1 N sodium hydroxide solution.

### 2.2. UV-VIS

**Figure 1:**
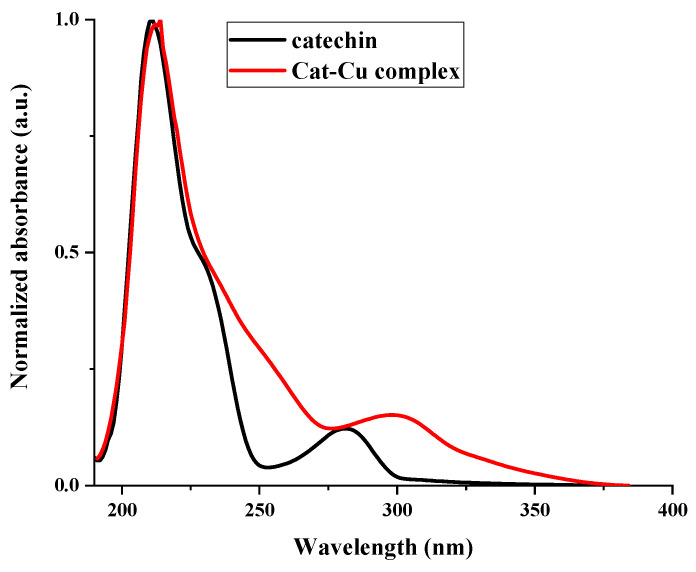
Comparative UV-VIS spectra of catechin and the cat–Cu complex.

The absorption spectra of the synthesized catechin–copper complex were measured using UV-VIS spectroscopy, as shown in Figure 1. The results indicated a significant shift in the absorption maximum of the complex when compared to that of catechin. This shift may serve as evidence that the complex was formed. The UV-VIS spectra provided insights into the mechanism underlying the formation of the catechin–copper bond. The presence of the copper ion facilitated a transition of the π → π* nature, leading to the formation of a condensed ring structure with catechin. This interaction involved the participation of the 3-OH and 4-oxo functional groups. The lack of the hydroxyl (OH) group in the complex can be ascribed to its reduced protonic acidity and the presence of steric hindrance. 

### 2.3. FT-IR

**Figure 2:**
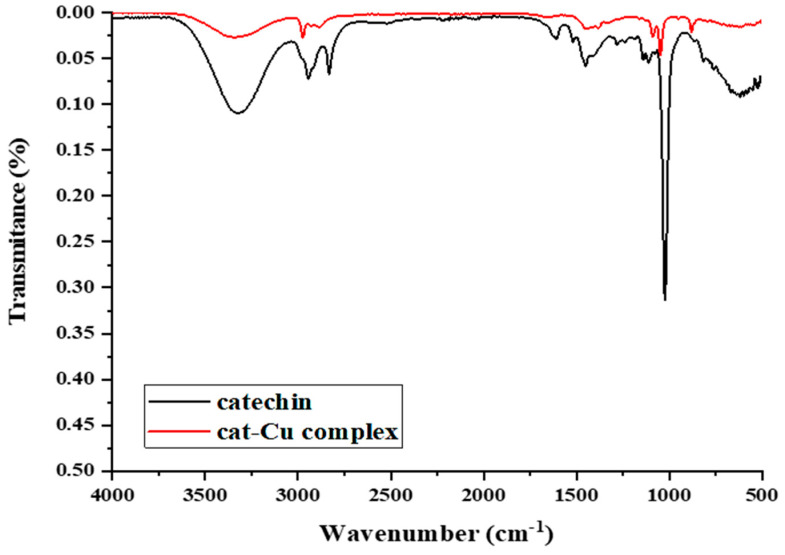
Comparative FT-IR spectra of catechin and the cat–Cu complex.

Upon binding with copper ion, the v(C=O) band of free catechin in the infrared spectrum exhibited a shift from 1657 cm^−1^ to 1598 cm^−1^ (Figure 2). This phenomenon can be attributed to the establishment of a coordination bond between the oxygen atom of the carbonyl group and the metal ions. 

The formation of a novel ring within the complex resulted in the amplification of the conjugative effect, leading to a noticeable shift in the particular infrared absorption band ν(C=C) from 1619 cm^−1^ to 1509 cm^−1^. The identification of bands at a wavenumber of 634 cm^−1^ in the complex indicated the existence of an oxygen–copper bond, which was absent in the pure catechin molecule.

**Figure 3:**
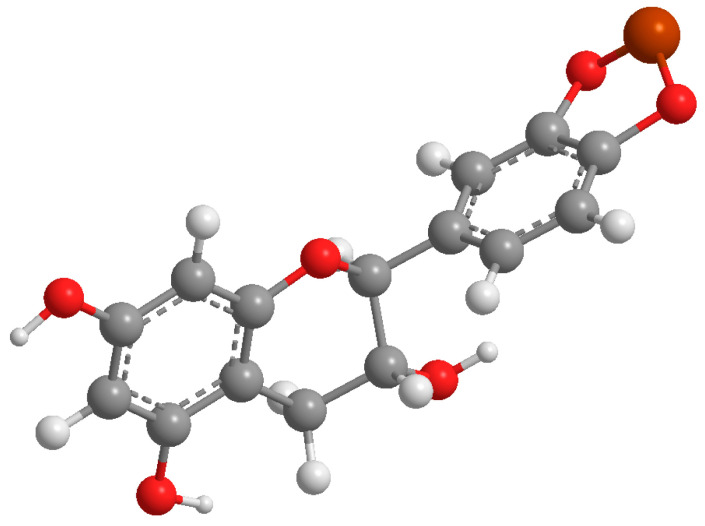
Proposed structure for the synthesized catechin–Cu(II) complex.

Figure 3 depicts the proposed structure for the catechin–Cu(II) complex based on the findings obtained from the FT-IR analysis. 

### 2.4. Morphological Analysis

**Figure 4:**
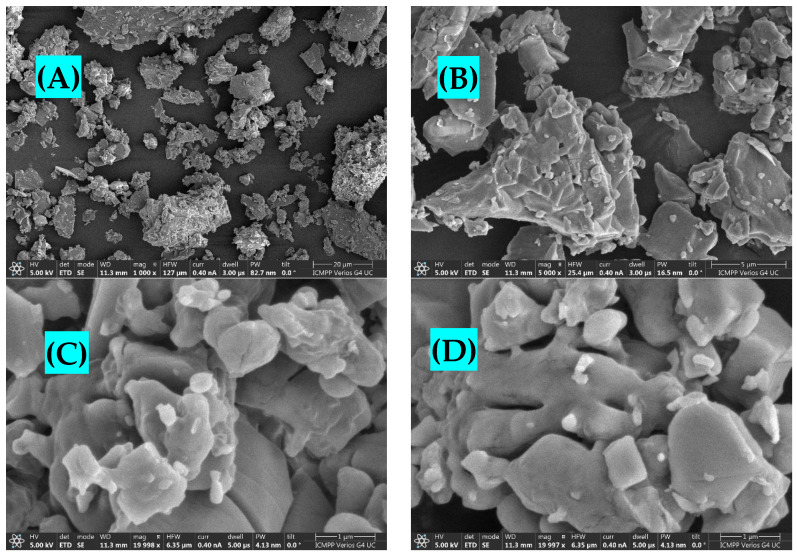
SEM images for the cat–Cu complex, (**A**) ×1000; (**B**) ×5000; (**C**) ×19,998; (**D**) ×19,997.

Scanning electron microscopy (SEM) was employed to capture images of the cat–Cu complex at various resolutions, such as 20, 5, 2, and 1 μm. The obtained images exhibit a fibrous morphology characterized by an acicular structure and an irregular fracture pattern (Figure 4). 

The present study documents the observation of the formation of minute clusters of crystals measuring 1 μm in diameter. These clusters exhibit adhesion at certain larger segments, potentially suggesting the presence of aggregation centers with varying degrees of complexity.

### 2.5. EDX

**Figure 5:**
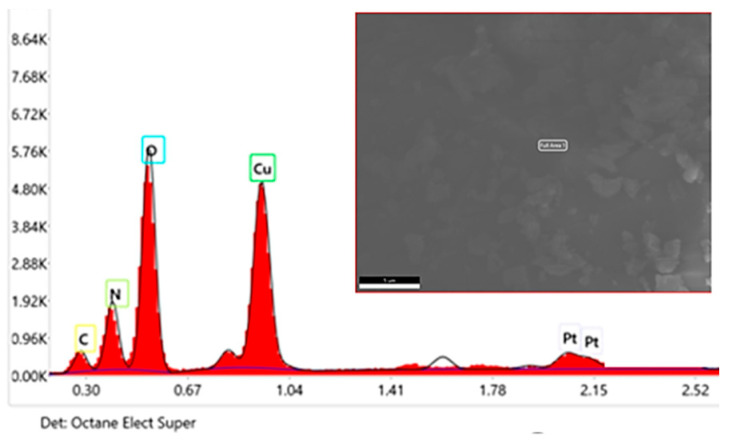
EDX of the synthesized cat–Cu complex.

The chemical composition and elemental presence within the chemical structure of the synthesized catechin–copper complex were confirmed through EDX analysis (Figure 5). Therefore, the presence of a copper content of 39.9% by weight was detected in the already purified and spectroscopically analyzed precipitate, thereby confirming the formation of the complex. These observations aligned with the data acquired through the utilization of FT-IR spectroscopy and UV-VIS spectroscopy.

The peaks registered by EDX analysis indicated a high concentration of copper in the sample. Figure 5 shows the analyzed area on a micron scale as well as the different concentrations of atoms by weight and by concentration. The EDX technique focuses mainly on the analysis of electrons found in the K layer of atoms.

### 2.6. In Vitro Antioxidant Activity

**Figure 6:**
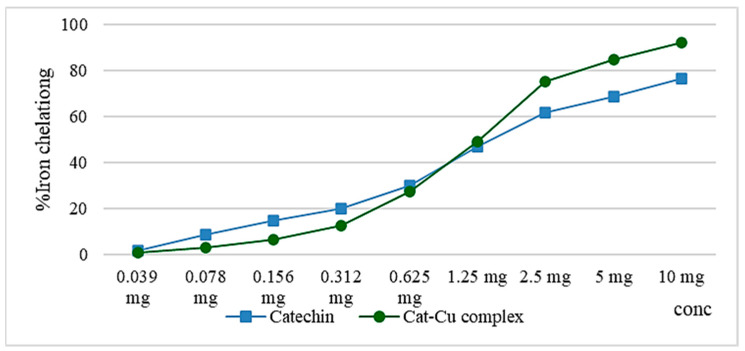
Graphical representation of the iron chelation capacity of the cat–Cu complex compared to that of catechin.

Copper, copper salt, and the cat–Cu complex have a higher ferrous ion chelation capacity compared to that of catechin, but comparing the data, there is the possibility of copper ions interfering with ferrozine that forms the colored complex with the Fe^2+^ ion. The preservation of catechin’s chelating ability post-complexation with copper ions offers antioxidant advantages via catechin itself, as well as through the copper ions, which can be utilized in the synthesis of superoxide dismutase, which is an enzyme responsible for the breakdown of the superoxide radical anion. Figure 6 shows the similar activity of the catechin and cat–Cu complex in terms of iron chelation capacity [7,13,14,15].

Catechin, by means of its hydroxyl groups, possesses the capability to chelate ferrous ions; however, this ability is inferior to that of substances with carbonyl groups, such as quercetin [12,16,17].

### 2.7. Lipoxygenase Activity

The assessment of lipoxygenase inhibition demonstrated that the complexation of catechin with copper ions significantly diminished the inhibitory impact, both relative to catechin alone and to copper ions alone. Research has indicated that the antioxidant properties of catechin are more effectively preserved when manufacturing nanoparticles with zinc oxide, as opposed to the catechin–zinc ion combination [3].

**Figure 7:**
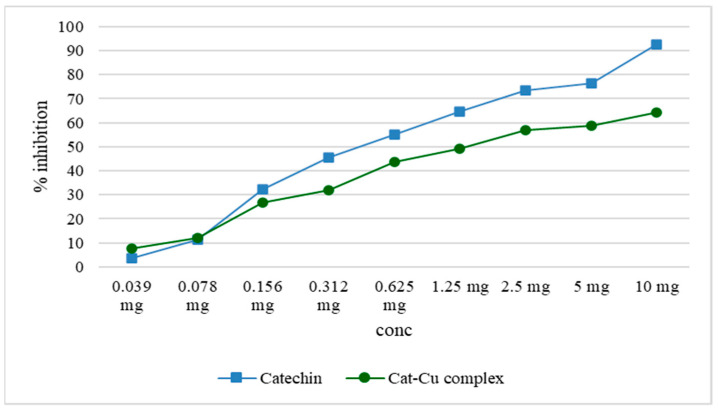
Graphic representation of the activity of the cat–Cu complex that reduces lipoxygenase activity.

Both catechin and the cat–Cu complex reduce lipoxygenase activity, reducing the amount of linoleic acid hydroperoxides (the substrate used for this determination). The inhibitory activity of the cat–Cu complex compared to that of catechin is shown graphically in Figure 7.

### 2.8. Determination of the Scavenger Capacity of Hydroxyl Radicals

Hydroxyl radicals are capable of altering the spatial structure of functional groups within protein structures, thereby impacting their biological function. This modification can lead to the development of pathological conditions such as atherosclerosis, neurodegenerative disorders, and tumor formation [18].

**Figure 8:**
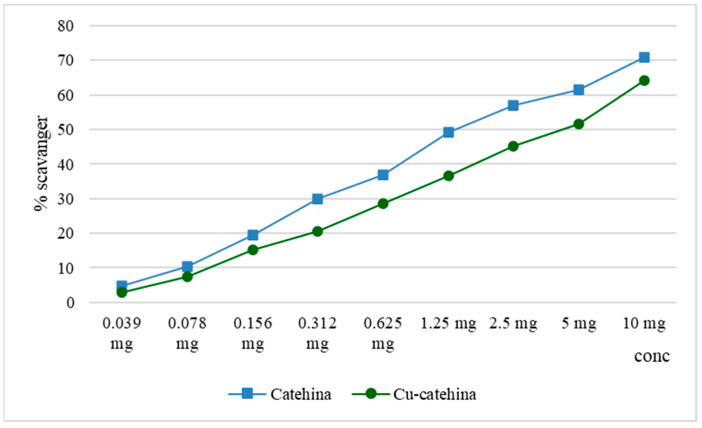
Hydroxyl radical scavenger activity of the cat–Cu complex.

In both the lipoxygenase inhibition test and the hydroxyl radical scavenger test, it was observed that the cat–Cu complex exhibited similar activity in comparison to catechin. This outcome can be attributed to the partial obstruction of the hydroxyl groups within the catechin structure. Hydroxyl groups, particularly those classified as phenolic, exhibit proton donor properties and have the capability to counteract the effects of free radicals. In contrast, the process of complexation demonstrates a more pronounced effect on the scavenging activity of zinc ions, resulting in a significant increase (*p* < 0.001). The results we obtained are displayed in Figure 8.

### 2.9. In Vitro Antidiabetic Activity

#### 2.9.1. Inhibition of Alpha-Amylase

The digestion of dietary starch requires pancreatic alpha-amylase and alpha-glucosidase, which convert starch into glucose molecules that are absorbed in the gut, enter the bloodstream, and induce postprandial hyperglycemia. The value is contingent upon the available starch for digestion, the activity of digestive enzymes, and the efficacy of intestinal absorption mechanisms. The elevation of blood sugar beyond physiological limits exacerbates oxidative stress and leads to uncontrolled protein glycosylation, hence aggravating diabetic symptoms and increasing the risk of organ problems [19,20,21].

**Figure 9:**
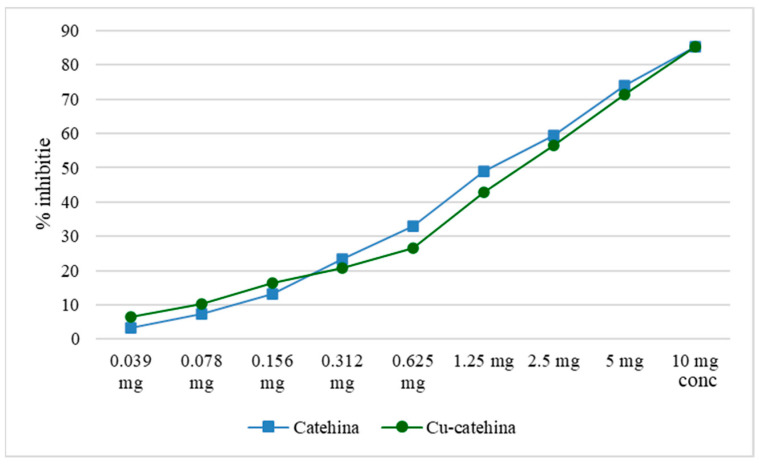
Alpha-amylase inhibition potential for the cat–Cu complex.

Metal ions inhibit or hinder enzyme function by altering its secondary structure, resulting in a higher frequency of beta-folded or linear configurations at the expense of alpha-helical structures [22]. Altering the secondary structure will impact the configuration of the enzyme’s active site and diminish its interaction with the substrate. Figure 9 illustrates the comparable inhibitory potential of alpha-amylase for the cat–Cu complex.

#### 2.9.2. Inhibition of Alpha-Glucosidase

According to Choudhary et al. (2020), the activity of alpha-glucosidase is diminished by catechin in a process in which it forms hydrogen bonds with the functional groups of amino acids present in the enzyme’s structure [7].

Numerous studies have shown that catechin and other vegetable-derived polyphenols can effectively block or reduce the activity of alpha-amylase and alpha-glucosidase enzymes. In certain instances, their effectiveness in this context exceeds that of acarbose, a medication frequently utilized to impede carbohydrate digestion. The inhibitory impact of copper on alpha-amylase or alpha-glucosidase is constant when delivered as nanoparticles, producing findings akin to those seen in the present work.

**Figure 10:**
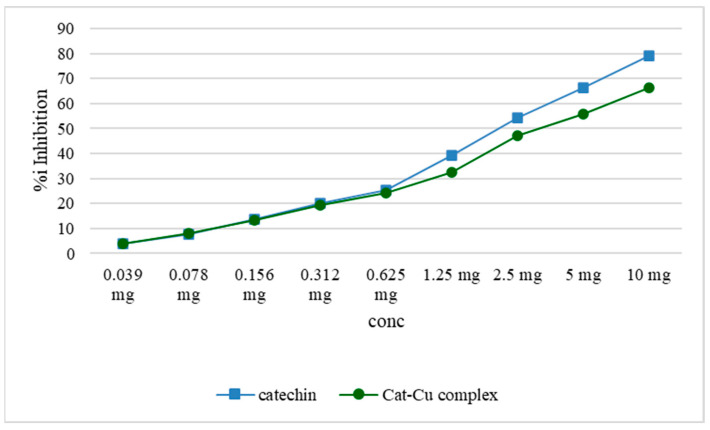
Graphic representation of alpha-glucosidase inhibition capacity of the cat–Cu complex compared to that of catechin.

Notably, a more pronounced impact on alpha-glucosidase is observed compared to alpha-amylase [23]. The comparative assay of catechin and the cat–Cu complex can be seen in Figure 10.

### 2.10. Antibacterial Activity

The evaluation of the antimicrobial activity expressed by the diameter of the inhibition zone was completed with the determination of the minimum inhibitory concentrations (MICs), the minimum bactericidal concentrations (MBCs), and fungicidal concentrations (MFC) on *Staphylococcus aureus* ATCC 25923, *Escherichia coli* ATCC 25922, *Pseudomonas aeruginosa* ATCC 27853, and *Candida albicans* ATCC 90028. Minimum inhibitory concentration (MIC) values are associated with the values of the lowest concentration of the synthesized and tested complexes that inhibited the growth of microbial cultures, compared to the positive control. We determined the minimum inhibitory concentrations (MICs) by the microdilution method in the broth for products with definite antimicrobial activity in the diffusometric method.

The synthesized cat–Cu complex exhibited modest antibacterial activity against *S. aureus* and modest antifungal activity against *Candida albicans*, and no antibacterial activity against *E. coli* and *P. aeruginosa* as can be seen in Table 2.

The evaluation of the antimicrobial activity, expressed by the diameter of the inhibition zone, was supplemented by determining the minimum inhibitory concentrations (MICs) and the minimum bactericidal concentrations (MBCs), as well as the minimum fungicidal concentrations (MFCs) against *S. aureus*, *Escherichia coli*, and *Candida albicans*.

The minimum inhibitory concentration (MIC) values represent the lowest concentration of the synthesized and tested complexes that inhibited the growth of microbial cultures, compared to the positive control. We determined the minimum inhibitory concentrations (MICs) using the broth microdilution method for the products with confirmed antimicrobial activity in the diffusion method.

The MBC/MFC values were determined by transferring 0.1 µL from each well that showed the complete inhibition of visible growth onto the surface of a solid medium plate. The subcultures were incubated for 24 h at 37 °C (for bacteria) and 30 °C (for fungi). The MBC/MFC value was considered the lowest concentration of the compound that destroyed 99.9% of the tested microorganisms.

The MICs and MBCs against *S. aureus* are presented in Table 3.

Many metals are known for their biocidal properties through different mechanisms of action, making them ideal for sterilizing surfaces or even as antibiotics. Copper is a potent antimicrobial agent, second to silver cations. The mechanism in which copper causes the apoptosis of bacteria is not perfectly defined but there are many theories to it [24,25,26]. 

Generally, copper cations enter bacteria by rupturing the membrane and being further carried by different proteins. The contact sterilization theory states that copper cations (I) and (II) accumulate on the outside of the pathogen; this accumulation causes a high-density charge which creates an imbalance in the outer bacterial space. This imbalance leads to the deformation of the cell causing the cell membrane to rupture and the contents to leak out [27,28].

Cu^2+^ + H_2_O_2_ → Cu^+^ + 2H^+^ + 2O^•^

Cu^2+^ + 2O^−^ → Cu^+^ + O_2_

Cu^2+^ + H_2_O_2_ → Cu^+^ + HO^•^ + HO^−^

The Fenton reaction is another way in which copper ions induce apoptosis; copper participates in redox reactions leading to the formation of ROS which in turn lead to oxidative stress and cellular death [29].

## 3. Materials and Methods

### 3.1. Starting Materials

Commercial starting materials were used as provided and all the solvents used for the reactions were spectrophotometric grade. (+)-catechin, copper sulphate, alpha-glucosidase, alpha-amylase, p-nitrophenyl-alpha-D-glucopyranoside (pNFG), sodium carbonate, starch, dinitrosalicilic acid, sodium hydroxide, iron sulphate, hydrogen peroxide, boric acid, linoleic acid, soy lipoxygenase, sodium acetate, ferrozine ethanol, methanol, DMSO, chloroform, and acetone were all provided by Sigma-Aldrich (Steinheim, Germany) and used without further purifications. Ciprofloxacin 10 µg (Oxoid) and nystatin 100 µg (HiMedia Laboratory, Einhausen, Germany) were used.

### 3.2. Synthesis of Cat–Cu Complex

One of the methods we used in order to obtain the catechin–copper complex was the use of catechin and copper sulphate in a 1:1 molar ratio. The catechin was dissolved in methanol under continuous stirring, then the copper salt was added gradually over 30 min, after which the reaction was maintained for 3 h at 40 °C under continuous stirring. Various systems were tried to adjust the pH, but the best results were obtained with 1 N NaOH at pH 8.5. The reaction was stopped and allowed to cool to room temperature. The solvent was removed by filtration, and the precipitate was washed several times with acetone and dried in an oven for 48 h at 40 °C to make sure all the residual solvent was removed, and the precipitate was dried to constant mass. The yielded complex thus obtained was further used for structural confirmation determinations, UV-VIS absorption spectroscopy, morphological analysis, and EDX.

### 3.3. Characterization of the Complex

- Instrumentation

The structural characterization of the individual reagents and reaction products was carried out by Fourier-transform infrared spectroscopy (FT-IR). The FT-IR spectra were registered on an FT-IR Bruker Vertex 70 spectrophotometer (Karlsruhe, Germany) by the ATR technique using samples as powders. The solubility of the synthesized complex was qualitatively evaluated by the dissolution of 5 mg of complex in 1 ml of several solvents (chloroform, acetone, ethanol, methanol, and DMSO). The UV-VIS spectra were recorded on a Shimadzu UV-1280 spectrophotometer (Duisburg, Germany) using a diluted polymer solution approx. 10^−5^ M in methanol. The morphology of the polymer coatings was evaluated by SEM using a Verios G4 UC Scanning Electron Microscope from Thermo Fisher Scientific (Waltham, MA, USA).

b. In Vitro Biological Activity Assay

For the newly synthetized complex, the investigation regarding the biological activity was assessed by the in vitro antioxidant, antidiabetic, and antibacterial activity. 

#### 3.3.1. Antioxidant Assay

Antioxidant potential was evaluated by three different methods: the iron chelation test, hydroxyl radical test, and inhibition of lipoxygenase (15-LOX) activity test [13,23,30]. 

For the iron (II) chelation test, ferrozine was used, due to its ability to form complexes with ferrous iron in a quantitative manner, resulting in the production of a pink color. Nevertheless, the introduction of chelating agents resulted in the disturbance of complex formation, which led to a decrease in the intensity of coloration. The monitoring of the ferrous ions was conducted through the measurement of the formation of a pink ferrous ion–ferrozine complex at a wavelength of 562 nm. The methodology employed in this study closely resembled the approach outlined by Burlec et al. [31]. Using 0.2 mL of sample solution in ultrapure water, 0.74 mL of 0.1 M acetate buffer solution (pH 5.25), 0.02 mL of 2 mM ferrous sulphate solution, and 0.2 M of hydrochloric acid were added, and after stirring for 10–15 s, 0.04 mL of 5 mM ferrozine solution was slowly poured. After 10 min of resting in the dark, the absorbance of the solution at 562 nm was determined using a standard solution, prepared under the same conditions as the sample (the ferrous sulphate solution was replaced by ultrapure distilled water). In parallel, the control solution and its blank were prepared, the control containing 0.2 mL of ultrapure water, 0.74 mL of 0.1 M acetate buffer solution (pH 5.25), 0.02 mL of 2 mM ferrous sulphate solution in 0.2 M hydrochloric acid, and after stirring for 10–15 s, 0.04 mL of 5 mM ferrozine solution [31,32].

The chelating capacity of the ferrous ion was calculated according to the following formula:

**activity % = 100 × (Ac − Ap)/(Ac)** (1)

where:

**Ac** represents the absorbance of the control solution;

**Ap** represents the absorbance of the sample solution.

The hydroxyl radical was generated through the reaction between the ferrous ion and hydrogen peroxide. This hydroxyl radical then reacts with salicylic acid, resulting in the formation of a pink–purple compound that exhibited its highest absorbance at a wavelength of 562 nm [5,13]. The absorbance of the control sample was measured at a wavelength of 562 nm, relative to the control sample where the ferrous sulfate solution was substituted with distilled water. In this experiment, a volume of 0.225 mL of sample solution dissolved in dimethyl sulfoxide (DMSO) was combined with 0.750 mL of a 1.5 mM iron (II) sulfate solution, 0.9 mL of a 20 mM sodium salicylate solution, and 0.525 mL of a 6 mM hydrogen peroxide solution. The mixture was incubated for 30 min at 37 °C. Subsequently, the mixture was allowed to cool down to the ambient room temperature. The absorbance of the sample (referred to as the control) was then measured at a wavelength of 562 nm. This measurement was compared to the control sample, where the ferrous sulfate solution was substituted with bi-distilled water. The positive control underwent the same processing conditions as the samples, with the exception that dimethyl sulfoxide (DMSO) was utilized in place of the sample solution [12,18,33].

The determination of lipoxygenase inhibition involved assessing the activity of 15-sLOX by spectrophotometrically monitoring the formation of reaction products at a wavelength of 234 nm. The experimental procedures involved conducting all reactions with a final volume of 2 mL and stirring the mixture using a magnetic bar at ambient temperature. The experimental setup involved the utilization of a reaction medium comprising a HEPES buffer with a concentration of 0.1 M and a pH value of 7.4. The experimental procedure involved the addition of the inhibitor (complex) in methanol to the cuvette containing the substrate buffer, followed by the subsequent addition of the enzyme [1,9].

An amount of 0.05 mL of 15-lipoxygenase solution in borate buffer pH 9 was treated with 0.05 mL of an analyte solution diluted in DMSO, and the mixture was left to rest for 10 min at room temperature, after which 2 mL of 0.16 mM linoleic acid solution in 0.1 M borate buffer pH 9 was added. The absorbance of the solution was recorded at 234 nm in the range of 0–120 s. In parallel, the positive control was processed in which the solution to be analyzed was replaced by DMSO. Gallic acid was used as a reference substance, and solutions of gallic acid in DMSO were processed under the same conditions as the methanolic extract.

All determinations were performed in triplicate, the results being expressed as the mean of three determinations ± standard deviation.

The lipoxygenase inhibition capacity was calculated according to the following formula:

**activity% = (AEFI − AECI) × 100/AEFI** (2)

where:

**AEFI** represents the difference between the absorbance of the enzyme solution without inhibitor at 90 s and the absorbance of the same solution at 30 s;

**AECI** represents the difference between the absorbance of the inhibitor-treated enzyme solution (sample) at 90 s and the absorbance of the same solution at 30 s.

#### 3.3.2. Antidiabetic Activity

An antidiabetic assay was performed by determining the alpha-amylase and alpha-glucosidase.

Alpha-amylase catalyzes the hydrolysis of starch with the release of glucose which reacts with dinitrosalicylic acid and forms a yellow–orange-colored compound with a maximum absorbance at 540 nm. In the presence of enzyme inhibitors, enzyme activity is blocked or reduced with a reduction in the absorbance of the solution at 540 nm. A 0.4 mL sample solution dissolved in DMSO was combined with 0.08 mL of an enzyme solution with a concentration of 2 IU/mL, 0.2 mL of a starch solution with a concentration of 0.5%, and 0.16 mL of a phosphate buffer with a concentration of 20 mM and a pH of 6.7. The resulting mixture was incubated at 37 °C 10 min. Following a time interval of 10 min, a volume of 0.32 mL of dinitrosalicylic reagent solution was introduced into the reaction mixture, which was subsequently subjected to a temperature of 100 °C for 15 min. The solution underwent a cooling process, following which the absorbance of the sample was measured relative to the sample control that lacked the addition of any enzyme. The positive control was acquired using the same procedure, employing DMSO instead of the complex [9,34,35,36].

The enzyme inhibition capacity was calculated according to the following formula:

**activity % = 100 × (Ac − Ap)/(Ac)** (3)

where:

**Ac** represents the absorbance of the control solution;

**Ap** represents the absorbance of the sample solution.

Alpha-glucosidase catalyzes the hydrolysis of pNFG to p-nitrophenylphosphate, a yellow compound with a maximum absorbance at 405 nm. In the presence of inhibitors, the enzyme activity decreases or is blocked with the reduction of absorbance of the solution at 405 nm [37,38].

We prepared the solutions following this protocol: 0.08 mL of 2 IU/mL enzyme solution, 0.2 mL of 0.5% starch, and 0.16 mL of 20 mM phosphate buffer solution pH 6.7 were added to 0.4 mL of sample solution in dimethyl sulfoxide and the solution was maintained for 10 min at 37 °C. After 10 min, 0.32 m of dinitrosalicylic reagent solution was added, and the reaction was maintained for 15 min at 100 °C. The solution was cooled, and the absorbance of the sample was read against the sample control in which no enzyme was added. The positive control was processed under the same conditions as the samples, but dimethyl sulfoxide was used instead of the sample solution.

The enzyme inhibition capacity was calculated according to the following formula:

**activity % = 100 × (Ac − Ap)/(Ac)** (4)

where:

**Ac** represents the absorbance of the control solution,

**Ap** represents the absorbance banks of the sample solution.

The IC50 value, expressed in µg sample/mL of final solution, was calculated for samples exhibiting an enzyme inhibition capacity exceeding 50%. The IC50 value was determined by considering the concentration of the antioxidant agent solution that corresponds to an activity of 50% using linear interpolation based on the first lower and higher values of 50%. The experiments were conducted in triplicate, and the outcomes were reported as the mean of three determinations ± standard deviation. In order to establish accurate correlations and determine statistical significance, a t-Student analysis was conducted.

#### 3.3.3. Antibacterial Activity

The antibacterial activity was determined using the disk diffusion test against *Staphylococcus aureus* ATCC 25923, *Escherichia coli* ATCC 25922, *Pseudomonas aeruginosa* ATCC 27853, *Klebsiella*, and *Candida albicans* ATCC 90028. 

We tested the antimicrobial activity of the synthesized complexes both by applying qualitative methods, through the diffusometric method, and by quantitative methods, applying the method of microdilutions in the broth (CLSI 2023, CLSI 2009). For the tests, the microorganisms were incubated in inclined tubes, with nutrient agar for bacteria and Sabouraud agar for fungi. 

A standardized inoculum of 5 × 10^4^ UFC/mL of the test bacterial strain was seeded in a discontinuous concentration gradient of the test products, made in Mueller–Hinton broth/Liquid Sabouraud medium. After overnight incubation at 37 °C, we determined the minimum inhibitory concentration as the lowest antibiotic dilution that inhibited visible bacterial growth.

The limits of the concentrations tested for each sample were between 0.01 and 10 mg/mL. The CMB/CMF values were determined by transferring 0.1 µL of each well that showed complete inhibition of visible growth onto the surface of a solid medium plate. The subcultures were incubated for 24 h at 37 °C (for bacteria) and 30 °C (for fungi). The CMB/CMF values were considered as the lowest compound concentration that killed 99.9% of the tested microorganisms.

The inoculum was obtained from the 24 h culture of each test microorganism and 48 h for *Candida* spp. The suspension in isotonic saline solution (0.9% NaCl) was adjusted to the density of the 0.5 McFarland standard, with the final density of ~108 Colony Forming Units/mL (CFU/mL) for bacteria and ~107 CFU/mL for *Candida* (CLSI, 2023). Mueller–Hinton agar medium was inoculated with this suspension, melted and brought to a temperature of 44–45 °C. The inoculated medium was distributed in Petri plates with a diameter of 9 cm in a volume of 10 mL/plate. For *Candida* spp. we used Sabouraud agar medium [10,39,40,41].

In stainless steel cylinders, with a diameter of 7 mm, placed on the surface of the medium inoculated with each test microorganism, we deposited with a micropipette a volume of 100 µL of each sample, after which the plates were incubated at 37 °C for bacteria and 30 °C for *Candida* spp. After 24–48 h of incubation, the antimicrobial activity was evaluated by measuring the diameter of the zone of inhibition of the growth of the test microorganisms compared to the control (reference standard) disks impregnated with the following precise concentrations of antibiotics: ciprofloxacin 10 µg (Oxoid) and nystatin 100 µg (HiMedia Laboratory) [42,43,44].

Each sample was tested 3 times, and the final result represented the average of the values of three diameters of the zones of the inhibition of the growth of test microorganisms.

## 4. Conclusions

Preserving or enhancing the antioxidant, enzymatic inhibitory, or antibacterial activities of catechin following complexation with metal ions may confer advantages for the application of these substances in preclinical research.

The justification for creating catechin–copper complexes is their ability to address the shortcomings of pure catechin in biomedical and pharmaceutical applications. Catechins are recognized for their antioxidant, anti-inflammatory, and antibacterial effects; nevertheless, their bioavailability and stability are generally inadequate, which can restrict their effectiveness. Catechin–copper complexes have many benefits: they augment the stability of catechins, safeguard them against fast breakdown, and raise their bioavailability. Copper is essential in biological activities, including enzymatic functioning and immunological responses, which may enhance the health advantages of catechins.

The aim of employing catechin–copper complexes in nutraceuticals is to formulate more potent, bioavailable, and stable compounds that can more effectively provide health benefits, including antioxidant protection, cardiovascular support, and improved antimicrobial activity, thereby surpassing the efficacy of pure catechins in functional foods and dietary supplements. The complexes can serve as green alternatives to preservatives and could be formulated as creams or gels for topical use in different afflictions such as skin cancer or burns. There are already patents for different copper ion-based creams for their use in skin cancer. 

Given the significance of copper ions in keeping insulin stability and the advantageous impact of the complex in decreasing carbohydrate digestion, these complexes have potential utility in the treatment of individuals with diabetes. The maintenance of physiological parameters related to oxidative processes in living organisms, such as the human body, frequently necessitates the assistance of endogenous antioxidant mechanisms, which can be supplemented by the intake of compounds known for their protective effects.

The assessment of the antioxidant potential of the synthesized cat–Cu complex revealed a similar activity in the antioxidant and antidiabetic capacity of catechin. Therefore, in order to preserve the antioxidative activity, it is imperative to optimize the metal–catechin complex extraction process or explore the potential utilization of catechin derivatives.

Consequently, it has become imperative to conduct in vivo toxicological and pharmacokinetic assessments to evaluate their suitability for therapeutic use.

## Figures and Tables

**Table 1:** Yields obtained by changing the pH and solvent.

Solvent	Water	Ethanol		Methanol		Ethanol 96		Hydroalcoholic Mix (1:1)	
**Yield (%)**	29.7	47.80		62.30		55.70		37	
pH	5	5.5	7	7.5	8	8.5	9	10	11
NaOH 1 N	-	-	63.1	62.30	67.80	70.2	69.2	69.50	65.20
HCL 1 N	8.5	7.3	-	-	-	-	-	-	-

**Table 2:** Comparing study of the diameter of inhibition for catechin, copper sulphate, and the cat–Cu complex for *S. aureus*, *E. coli*, *P. aeruginosa*, and *Candida albicans*.

Tested Substance	*S. aureus* ATCC 25923	*E. coli* ATCC 25922	*P. aeruginosa* ATCC 27853	*C. albicans* ATCC 90028
**Catechin**	20.0 ± 0.00	0	16.0 ± 0.00	0
**Copper sulphate**	14.0 ± 0.00	0	0	0
**cat–Cu complex**	17.3 ± 0.57	0	0	17.0 ± 0.00
**blank (DMSO)**	0	0	0	0
**I. Ciprofloxacin (5 µg/disc)**	30.0 ± 0.00	34.0 ± 0.00	31.3 ± 0.57	Not tested
**I. Fluconazol (25 µg/disc)**	Not tested	Not tested	Not tested	30.0 ± 0.00

**Table 3:** The MICs and MBCs of the synthesized complex and individual reagents against *S. aureus*.

Sample	*S. aureus* ATCC 25923	
	MIC (mg/mL)	MBC (mg/mL)
Catechin	1.25	2.5
Copper sulphate (II)	1.25	2.5
Catechin–Cu (II)	0.07	0.15

## Data Availability

The original contributions presented in the study are included in the article, further inquiries can be directed to the corresponding author/s.
